# Factors related to parenting difficulties and depression in the COVID‐19 pandemic

**DOI:** 10.1002/kjm2.12820

**Published:** 2024-03-19

**Authors:** Ching‐Shu Tsai, Ray C. Hsiao, Cheng‐Fang Yen

**Affiliations:** ^1^ Department of Child and Adolescent Psychiatry Chang Gung Memorial Hospital, Kaohsiung Medical Center Kaohsiung Taiwan; ^2^ School of Medicine Chang Gung University Taoyuan Taiwan; ^3^ Department of Psychiatry Seattle Children's Seattle Washington USA; ^4^ Department of Psychiatry and Behavioral Sciences School of Medicine, University of Washington Seattle Washington USA; ^5^ Department of Psychiatry School of Medicine College of Medicine, Kaohsiung Medical University Kaohsiung Taiwan; ^6^ Department of Psychiatry Kaohsiung Medical University Hospital Kaohsiung Taiwan; ^7^ College of Professional Studies National Pingtung University of Science and Technology Pingtung Taiwan

Dear Editor,

Parents have encountered multidimensional stressors when caring for their children during the coronavirus disease 2019 (COVID‐19) pandemic.^1^ Such parenting challenges have been reported to be associated with parental distress and mental health problems.^2^ These findings underscore the necessity of further investigating parenting difficulties among parents of children during the COVID‐19 pandemic. Identifying the associations of demographic characteristics and fear of COVID‐19 with parenting difficulties and depression in the COVID‐19 pandemic can help develop group‐specified intervention programs for addressing the mental health of parents.

This study enrolled 550 parents (429 women and 121 men; mean age = 43.78 ± 5.23 years; mean years of education = 16.28 ± 2.48 years; 509 married and 41 divorced) through posting an online advertisement on Facebook, LINE, and the Professional Technology Temple bulletin board system from August 2022 to April 2023. The mean age of their children (302 boys and 248 girls) was 11.33 (SD = 3.57) years. Parents' self‐reported parenting difficulties during the COVID‐19 pandemic were assessed using the Parenting Difficulties in Infectious Disease Pandemic Inventory (PDIDPI).^3^ The present study transformed the seven factor scores of the PDIDPI into a factor score of parenting difficulties using factor analysis; the transformed mean score was 0 (SD = 1; ranging from −1.78 to 3.39). Parents' depressive symptoms and the fear of COVID‐19 were assessed using the Center for Epidemiologic Studies Depression Scale (mean = 11.37; SD = 7.10; ranging from 0 to 36)^4^ and Fear of COVID‐19 Scale (mean = 14.73; SD = 5.21; ranging from 6 to 30),^5^ respectively. The factors related to parenting difficulties and parental depression were examined using multivariate linear regression analysis. Because of the high correlation between parent's and child's age (Pearson's *α* coefficient = 0.528), only child's age entered into the regression model.

The results found that caring a boy (*p* = 0.041), caring for a younger child (*p* < 0.001), and greater fear of COVID‐19 (*p* < 0.001) were significantly associated with greater parenting difficulties in the COVID‐19 pandemic (Table 1). Furthermore, being divorced (*p* = 0.041), greater fear of COVID‐19 (*p* < 0.001), and greater parenting difficulties (*p* < 0.001) were significantly associated with greater parental depression in the COVID‐19 pandemic.

**TABLE 1:** Factors related to parenting difficulties and depression: multivariate linear regression.

	Parenting difficulties		Parental depression	
	B (SE)	*p*	B (SE)	*p*
Parent's sex^a^	0.006 (0.100)	0.954	0.089 (0.654)	0.891
Parent's marriage status^b^	0.199 (0.159)	0.211	2.120 (1.037)	0.041
Parent's educational year	−0.002 (0.017)	0.894	−0.055 (0.111)	0.624
Child's sex^c^	0.170 (0.083)	0.041	−0.585 (0.544)	0.283
Child's age	−0.046 (0.012)	<0.001	−0.034 (0.078)	0.666
Parent's fear of COVID‐19	0.037 (0.008)	<0.001	0.372 (0.053)	<0.001
Parenting difficulties			2.293 (0.280)	<0.001
*F*	7.288		22.055	
*p*	<0.001		<0.001	
Adjusted *R* ^2^	0.064		0.219	

^a^ Female as the reference.

^b^ Being married as the reference.

^c^ Girls as the reference.

Parenting difficulties and parental depression are the results of interactions of parents with their children and environments. The levels of parenting difficulties and parental depression were high in groups of parents of boys and younger children, indicating that intervention programs should help parents to develop the parenting skills for children of various sex and age. Divorced parents had a higher level of depression compared with married parents, indicating that divorced parents may need further support during the COVID‐19 pandemic. The fear of COVID‐19 is the response pertaining to the threat of COVID‐19. Although the fear generally drives actions toward self‐protection, it may also aggravate parents' psychological burden, parenting difficulties and subsequently depression. There is a need to help parents to develop effective coping strategies to reduce the fear of COVID‐19.

## CONFLICT OF INTEREST STATEMENT

The authors declare no conflicts of interest.

## ETHICS STATEMENT

All participants provided informed consent before undergoing any assessments. This study was approved by the Institutional Review Board of Kaohsiung Medical University Hospital (KMUHIRB‐KMUHIRB‐E(I)‐20220107) and Chang Gung Medical Foundation (202200544B0C501).
